# Effect of Various Inoculum Levels of Multidrug-Resistant *Salmonella enterica* Serovar Heidelberg (2011 Ground Turkey Outbreak Isolate) on Cecal Colonization, Dissemination to Internal Organs, and Deposition in Skeletal Muscles of Commercial Turkeys after Experimental Oral Challenge

**DOI:** 10.3389/fmicb.2017.02680

**Published:** 2018-01-12

**Authors:** Divek V. T. Nair, Jijo Vazhakkattu Thomas, Sally Noll, Robert Porter, Anup Kollanoor Johny

**Affiliations:** ^1^Department of Animal Science, University of Minnesota, Saint Paul, MN, United States; ^2^Department of Veterinary Population Medicine, University of Minnesota, Saint Paul, MN, United States

**Keywords:** *Salmonella* Heidelberg, turkeys, challenge, colonization, dissemination, muscle, multidrug-resistant

## Abstract

*Salmonella enterica* serovar Heidelberg (*S*. Heidelberg) is a major foodborne pathogen colonizing poultry. The pathogen is associated with a significant number of foodborne outbreaks through contaminated poultry meat, including turkeys. Recently, multidrug-resistant (MDR) strains of *S*. Heidelberg have emerged as a threat to human public health in the United States. The objective of this study was to determine the cecal colonization, dissemination to internal organs, and the potential for skeletal muscle deposition of an MDR *S.* Heidelberg isolate from the 2011 ground turkey outbreak in the United States after the experimental oral challenge of poults (young turkeys) and adult turkey hens. In the poult study, two separate experiments using day-old, straight-run, commercial hybrid converter poults were randomly assigned to five challenge groups (0, 10^∧^2, 10^∧^4, 10^∧^6, 10^∧^8 CFU groups; 12 poults/group; *N* = 60/experiment) and a week after, treatment groups were challenged separately with 0-, 2-, 4-, 6-, and 8- log_10_ CFU of *S.* Heidelberg orally. After 14 days post-challenge, the poults were euthanized, and samples were collected to determine MDR *S.* Heidelberg colonization in the cecum, dissemination to liver and spleen, and deposition in the thigh, drumstick, and breast muscles. A similar experimental design was followed for the adult turkey hens. In two separate experiments, 11-week-old commercial Hybrid Converter turkey hens (4 hens/group; *N* = 20/experiment) were challenged with MDR *S*. Heidelberg and on day 16 post-challenge, birds were euthanized and samples were collected to determine *Salmonella* populations in the samples. The results indicated that, in turkey poults, the recovery of MDR *S.* Heidelberg was highest in the cecum followed by spleen, liver, thigh, drumstick, and breast. All tested inoculum levels resulted in more than 3.5 log_10_ CFU/g colonization in the poult cecum. The cecal colonization, dissemination to internal organs, and tissue deposition of MDR *S.* Heidelberg were high in poults. The pathogen recovery from the cecum of adult turkey hens ranged from 37.5 to 62.5% in the challenge groups. The results signify the importance of controlling MDR *S*. Heidelberg in turkeys at the farm level to improve the safety of turkey products.

## Introduction

Gastrointestinal (GI) illness caused by foodborne pathogens is a major public health concern resulting in significant loss to the United States economy (Scallan et al., 2011; Scharff, 2012; Marder et al., 2017). Poultry meat and eggs contribute to >50% foodborne outbreaks associated with non-typhoidal *Salmonella* (CDC, 2013). Poultry, including turkeys, are a common reservoir host for *Salmonella* and are commonly implicated contamination vehicles for human infections (Mead et al., 2010). The colonization of the GI tract of poultry with the pathogen results in the excretion through droppings, subsequently contaminating the farm environment and the poultry carcasses during processing. Prevalence of *Salmonella* in poultry-derived foods, including turkeys, along with increased consumer taste for poultry products in the United States are potential risk factors for foodborne outbreaks (Foley et al., 2008; NCC, 2017).

*Salmonella* has > 2500 serovars, and among these, 7% are associated with foodborne outbreaks through poultry. The predominant serovars such as *S.* Enteritidis and *S.* Typhimurium are continuing as major pathogens whereas emerging serovars such as *S.* Heidelberg, *S*. Infantis, and *S*. Oranienburg are new threats to the United States food industry (Foley et al., 2011; CDC, 2016; Hindermann et al., 2017). Among these, *S.* Heidelberg is highly invasive affecting humans and is among the top five *Salmonella* serovars frequently associated with human salmonellosis (CDC, 2013). In addition, *S.* Heidelberg is among the top three serovars of *Salmonella* commonly isolated from chickens under pathogen reduction and HACCP (PR: HACCP) verification samples for broiler meat and outbreaks associated with chickens (FSIS, 2010; Finstad et al., 2012). Moreover, *S.* Heidelberg is commonly isolated from turkey production facilities and has been accountable for 14% of foodborne outbreaks through turkeys in the previous years (1998–2008) (Jackson et al., 2013). Furthermore, *S.* Heidelberg is only second to *S.* Enteritidis in causing foodborne outbreaks through eggs (Jackson et al., 2013).

Development of antibiotic resistance in *S.* Heidelberg and the involvement of resistant strains in foodborne outbreaks through poultry is a serious concern. In 2010, 65% *S.* Heidelberg isolated from ground turkey were resistant to multiple drugs, including ceftriaxone, the drug of choice for treating human salmonellosis, and many other clinically relevant antibiotics such as streptomycin, tetracycline, sulfamethoxazole, chloramphenicol, and trimethoprim-sulfamethoxazole (Hoffmann et al., 2012, 2014). In 2011, *S*. Heidelberg caused a foodborne outbreak through contaminated ground turkey products resulting in 136 reported cases from 34 states. Some of the isolates implicated in the outbreak were resistant to common antibiotics such as ampicillin, streptomycin, gentamicin, and tetracycline. These isolates carry five plasmid-encoded resistance genes such as *bla*TEM-1, *aac (3)-IIa*, *aadA1*, *ant (3*_*)-Ia*, and *tetA.* The encoded plasmids are the IncI1 type that are common poultry associated plasmids (Folster et al., 2012). In addition, foodborne outbreaks linked to multidrug-resistant (MDR) *S.* Heidelberg were reported from poultry products from a leading producer in California and to the mechanically separated chicken from a Tennessee correctional facility in 2013 and 2014, respectively (CDC, 2014a,b). Moreover, Rothrock et al. (2015) reported the recovery of MDR *S.* Heidelberg from water used in scalding tanks, underscoring the necessity of understanding this pathogen as a public health threat emerging from the poultry farms.

Although the current literature presents some evidence on the use of antibacterials against *S*. Heidelberg, since the 2011 ground turkey outbreak, studies that determine the response of turkeys to the MDR clones of this pathogen, are only emerging. The objectives of the current study were to determine (1) the colonization potential of a 2011 ground turkey outbreak isolate in poults (young turkeys) and adult turkey hens, and (2) the dose required for effective cecal colonization, dissemination of the pathogen to liver and spleen, and the risk of deposition in skeletal muscle tissues, after the experimental oral challenge.

## Materials and Methods

All animal experiments were approved by the Institutional Animal Care and Use Committee, and the use of MDR *S*. Heidelberg (infectious agent) in turkeys was approved by the Institutional Biosafety Committee at the University of Minnesota.

### Pathogen, Growth Conditions, and Inoculum Preparation

One of the 2011 ground turkey outbreak isolates of MDR *S.* Heidelberg was used in the current study (Donators – Dr. Irene Hanning, College of Genome Sciences and Technology, University of Tennessee, and Dr. Kumar Venkitanarayanan, University of Connecticut; Identity of the isolate at Dr. Kollanoor Johny’s lab: GT2011). The glycerol stocks of MDR *S.* Heidelberg was prepared and stored at -80°C before the experiment. Working cultures were prepared by transferring 100 μl of MDR *S.* Heidelberg from glycerol stock to 10 ml tryptic soy broth (TSB; catalog no. C7141, Criterion, Hardy Diagnostics, Santa Maria, CA, United States) and were incubated at 37°C for 24 h with agitation (100 rpm). For selective enumeration, the pathogen was made resistant to 50 μg/ml nalidixic acid sodium salt (NA; CAS. no. 3374-05-8, Alfa Aesar, Haverhill, MA, United States). Growth of NA resistant *S.* Heidelberg (Identity of the isolate at Dr. Kollanoor Johny’s lab: GT2011NAL) in overnight broth cultures (24 h) was determined by plating appropriate dilutions of *S.* Heidelberg on xylose lysine desoxycholate agar plates (XLD; catalog no. C 7322, Criterion, Hardy Diagnostics, Santa Maria, CA, United States) containing 50 μg/ml NA and incubating at 37°C for 24 h. For inoculating birds, *S.* Heidelberg was grown in 100 ml TSB containing 50 μg/ml NA. The pathogen inoculum was prepared from 24 h broth culture after centrifuging at 3,600 ×*g* for 15 min at 4°C and resuspending the pellets in phosphate-buffered saline (PBS; pH 7.2). Five different inoculum levels of *S.* Heidelberg: 0, 10^∧^2, 10^∧^4, 10^∧^6, and 10^∧^8 CFU/ml were used in poults and adult turkey hens, in separate studies.

### Experimental Design

#### Turkey Poult Study

Day-old poults were purchased from a commercial turkey hatchery in Minnesota and were housed in the BSL2 Veterinary Isolation Facility of the Research Animal Resources (RAR) at the University of Minnesota. Two separate experiments were conducted. In each experiment, 60 poults were randomly distributed to 5 treatment groups (12 poults/treatment group): a negative control (Negative Control; 0 CFU *S.* Heidelberg), challenge group 1 (2-log; 10^∧^2 CFU *S.* Heidelberg), challenge group 2 (4-log; 10^∧^4 CFU *S.* Heidelberg), challenge group 3 (6-log; 10^∧^6 CFU *S.* Heidelberg) and challenge group 4 (8-log; 10^∧^8 CFU *S.* Heidelberg). On day 0, the incoming flock was tested for any inherent *Salmonella* by enriching the fecal samples in Selenite Cysteine Broth (SCB, Hardy Diagnostics; *n* = 6). On day 7, the poults were challenged with appropriate levels of MDR *S.* Heidelberg as crop gavage. *S.* Heidelberg recovery was determined in the cecum (for colonization), spleen and liver (for dissemination), and skeletal muscles [drumstick (*Peroneus longus*), thigh (*Semimembranosus*) and breast (*Pectoralis major*)] for potential muscle deposition, after euthanizing poults on days 9 (2 poults/group) and 21 (10 poults/group) of the study. *S.* Heidelberg colonization was ensured on day 9. Tissue samples (liver, spleen, and muscles; 2 samples/study) were also collected on day 21 for histopathology and immunohistochemistry examination.

#### Turkey Hen Study

Adult turkey hens (11-weeks old) were housed in the BSL2 Veterinary Isolation Barn of the RAR at the University of Minnesota. Two separate experiments were conducted. In each experiment, 20 hens were randomly distributed to 5 treatment groups (4 hens/treatment group) as mentioned for the poult study (Negative Control, 2-log, 4-log, 6-log, and 8-log). Turkeys were given a week for acclimatization in the isolation barn pens and were tested for any inherent *Salmonella* by enriching the fecal samples (*n* = 6). A week after, the birds were challenged with appropriate levels of MDR *S.* Heidelberg orally. *S.* Heidelberg recovery rates were determined in the cecum, spleen, liver, and skeletal muscles after euthanizing birds on day 16 post-challenge (4 hens/group) of the study. Tissue samples (liver, spleen, and muscles; 4 samples/group) were collected for histopathology and immunohistochemistry examination.

### Determination of *S.* Heidelberg in Cecum, Liver, Spleen, and Skeletal Muscles

The samples were collected in 50 ml sterile PBS tubes on the day of *S.* Heidelberg recovery (necropsy days). Samples were homogenized, 10-fold serially diluted in PBS and 200 μl of appropriate dilutions were surface plated on XLD + NA plates. *S.* Heidelberg enumeration was conducted after incubating the plates at 37°C for 24 h. In addition, all samples from the poult and adult turkey hen studies were enriched in 10 ml SCB on the day of collection. After incubation for 8–12 h at 37°C, cultures from SCB was streaked on XLD and XLD + NA plates. The plates were incubated for 24 h at 37°C to detect the presence of *S.* Heidelberg in the enriched samples (Hammack et al., 1999). In adult turkey hens, the fecal samples were also enriched daily for 16 days post-challenge to detect fecal shedding of *S.* Heidelberg. This step was to determine if the adult turkey hens remained positive for the pathogen despite their maturity.

### Histopathology Examination

Histopathological examination of tissue samples from liver, spleen, and muscles [drum stick (*Peroneus longus*), thigh (*Semimembranosus*) and breast (*Pectoralis major*)] of the birds was conducted. Tissue sections of 5 mm thickness were collected in 10% neutral buffered formalin from both challenged and non-challenged groups. Histological examinations were carried out after processing and staining the samples using a standard hematoxylin and eosin staining (Gu et al., 2015; Tavakkoli et al., 2015) at the Veterinary Diagnostic Laboratory at the University of Minnesota.

### Immunohistochemistry

The formalin-fixed tissue samples were also used for detection of *Salmonella* antigens in the tissues using antibodies specific for *Salmonella* by conducting immunohistochemistry as described previously (Campero et al., 2002). Briefly, the paraffin-embedded sections of tissues were deparaffinized by placing in a slide rack at 60–70°C for 30–45 min. Then the tissue sections were rehydrated by passing through descending grades of alcohol in a chemical hood. After washing, the slides were treated with Proteinase K (Dako Agilent Pathology Solutions, Santa Clara, CA, United States) enzyme for the retrieval of antigen and incubated in a humidity chamber for 5 min. The slides were then immersed in 0.05M TBS/Tween 20 buffer after washing with distilled water. The endogenous peroxidase enzyme was blocked by adding 3.0% H_2_O_2_ and incubated for 15 min. Then the slides were incubated adding mouse anti-*Salmonella* LPS core antibody (ViroStat, Inc., Portland, ME, United States) for 45 min at room temperature. Positive and negative samples were included. After incubating with primary antibody, the slides were incubated for 45 min at room temperature with goat anti-mouse IgG (H + L), HRP conjugate (Dako Agilent Pathology Solutions, Carpinteria, CA, United States) which served as secondary antibody. A chromogen, 3-Amino-9-Ethylcarbazole (Dako Agilent Pathology Solutions, Carpinteria, CA, United States) was added to the slides and incubated for 15 min to detect the immune reactivity. The slides were washed and stained with Mayer’s Hematoxylin (counterstain) for 5 min. The slides were then rinsed with tap water, mounted and observed under the microscope.

### Statistical Analysis

Data from the poult and hen studies were evaluated separately due to the difference in the colonization of *S*. Heidelberg in different age groups. A completely randomized design with a 2X5X6 factorial treatment structure was used for both studies. The factors included two experiments/study, five inoculum levels (0, 10^∧^2, 10^∧^4, 10^∧^6, and 10^∧^8 CFU/ml), and six tissue samples (cecum, liver, spleen, breast, thigh, drumstick). An isolator (poult study) or an isolator room (adult turkey hen study) was the experimental unit, and each study were repeated (two experiments per age group; a total of four experiments discussed in the manuscript). The number of birds used in the study was sufficient to detect significant mean differences in the pathogen counts between the groups with a two-sided alpha = 0.05 and statistical power of 0.99 and 0.91, respectively, for the poult and hen studies. Normally distributed data were analyzed using the PROC-MIXED procedure of the SAS software (version 9.4, SAS Institute, Cary, NC, United States). Whenever a qualitative analysis was required (presence and absence), we used PROC-GENMODE procedure of the SAS software. A *P* value of 0.05 was considered statistically significant. For histopathology and immunohistochemistry, presence or absence of bacteria by visual analysis was carried out (Gonzalez-Escobedo et al., 2013).

## Results and Discussion

Non-typhoidal *Salmonella* is the leading bacterial cause of foodborne illness in the United States. Poultry and poultry products, including turkeys, are epidemiologically linked to human outbreaks and remain major contributors of foodborne infections. Although carcass processing plays a significant role in the cross-contamination, farms are the focal points of *Salmonella* persistence and distribution to poultry. Several sources such as litter, feed, water, transportation equipment and vectors including insects, humans, and rodents exist on farms (Jones et al., 1991; Suzuki, 1994; Hoover et al., 1997; Foley et al., 2008). In addition, poultry serves as the natural reservoir host for several *Salmonella* serovars (Hopper and Mawer, 1988; Foley et al., 2011; Kollanoor Johny and Kumar, 2016). *Salmonella* enters the intestinal tract of poultry after ingestion and establishes colonization in the cecum. The pathogen uses various virulence mechanisms to cross the intestinal barriers, multiplies in the reticuloendothelial system and invades liver, spleen, ovary, and oviduct resulting in the systemic spread of the infection (Suzuki, 1994; Kollanoor-Johny et al., 2012a,b; Kollanoor Johny and Kumar, 2016). In the current study, *S.* Heidelberg was selected since it is an emerging serovar of *Salmonella* that contributes to human infections and *Salmonella*-related deaths in the United States (Kennedy et al., 2004; Gokulan et al., 2013). In addition, there is a recent interest in studying the MDR strains of *S*. Heidelberg involved in the 2011 ground turkey outbreak (Bearson et al., 2017; Nair and Kollanoor Johny, 2017). In the present study, different inoculum levels of *S.* Heidelberg were administered in poults and adult turkey hens orally. Then the colonization efficacy of the MDR isolates in the cecum, dissemination to liver and spleen, and potential risk of the pathogen deposition in the muscle tissues [drum stick (*Peroneus longus*), thigh (*Semimembranosus*) and breast (*Pectoralis major*)] were studied.

### Turkey Poult Study

**FIGURE 1:**
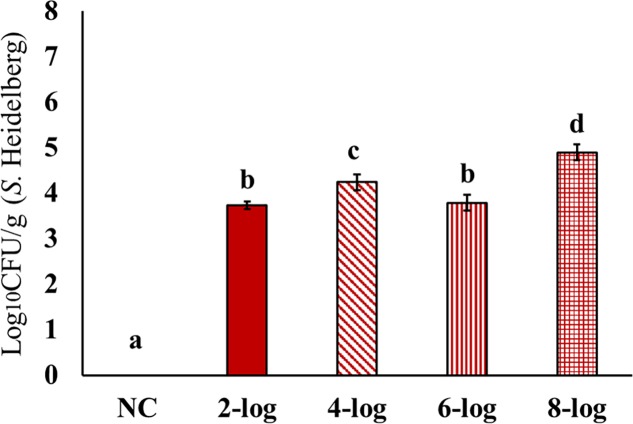
Effect of different inoculum levels on MDR *S.* Heidelberg colonization in the ceca of poults (Means ± SE; NC, 2-log, 4-log, 6-log, and 8-log treatments had a total of 20, 20, 18, 19, and 20 cecum samples/group, respectively, for the final analysis. ^a-d^ Bars with different superscripts differ significantly from each other at *P* < 0.05). NC, Negative Control.

*Salmonella* Heidelberg showed high colonization potential in the cecum of poults resulting in an efficient colonization for all the tested inoculum levels (2-, 4-, 6-, and 8- log) (**Figure 1**). All cecal samples tested positive for *Salmonella*. In both experiments, 2-log, 4-log, and 6-log of MDR *S.* Heidelberg resulted in 3.7–4.9 log_10_ CFU/g colonization in the cecum of poults. However, 8-log inoculum resulted in the maximum colonization among the tested inoculum levels (*P* ≤ 0.05; 5.1 and 4.7 log_10_ CFU/g in experiments 1 and 2, respectively). The results indicated that the inoculation level as low as 2-log is capable of effective colonization of MDR *S*. Heidelberg in the cecum of poults (**Figure 1**).

**FIGURE 2:**
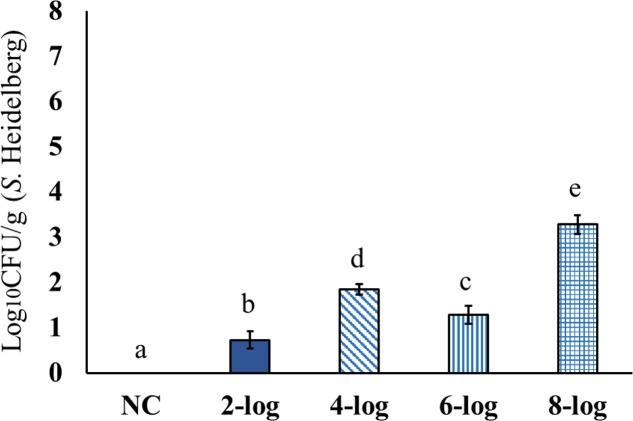
Effect of different inoculum levels on the dissemination of MDR *S.* Heidelberg to spleen of poults (Means ± SE; NC, 2-log, 4-log, 6-log, and 8-log treatments had a total of 18, 18, 16, 17, and 18 spleen samples/group, respectively, for the final analysis. ^a-e^ Bars with different superscripts differ significantly from each other at *P* < 0.05). NC, Negative Control.

**FIGURE 3:**
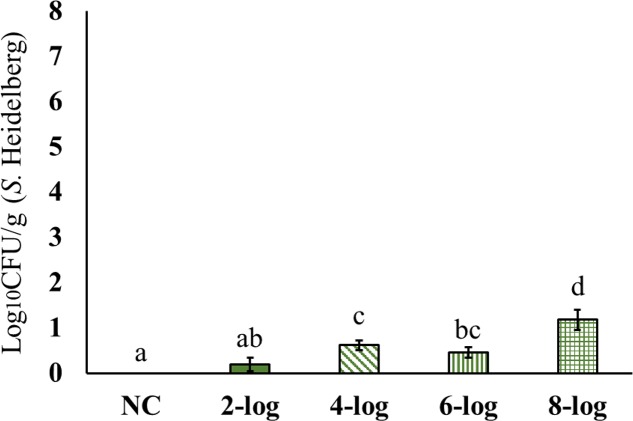
Effect of different inoculum levels on the dissemination of MDR *S.* Heidelberg to liver of poults (Means ± SE; NC, 2-log, 4-log, 6-log, and 8-log treatments had a total of 20, 20, 18, 18, and 20 liver samples/group, respectively, for the final analysis. ^a-d^ Bars with different superscripts differ significantly from each other at *P* < 0.05). NC, Negative Control.

Results showed significant dissemination of MDR *S*. Heidelberg to spleen for all the tested inoculum levels (**Figure 2**). The highest counts were observed with 8-log inoculum of *S.* Heidelberg that resulted in ∼3 log_10_ CFU/g in the spleen of 21 days old poults. The 4-log, and 6-log inoculation levels resulted in 1.0 to 2.0 log_10_ CFU/g *S*. Heidelberg in the spleen (**Figure 2**). However, dissemination of MDR *S.* Heidelberg to the liver was less compared to that of the spleen (**Figure 3**). Majority of the spleen (61 positive/69 total), and liver (46 positive/76 total) samples were positive by surface plating. Samples that yielded no colonies by surface plating were confirmed negative by enrichment method.

**FIGURE 4:**
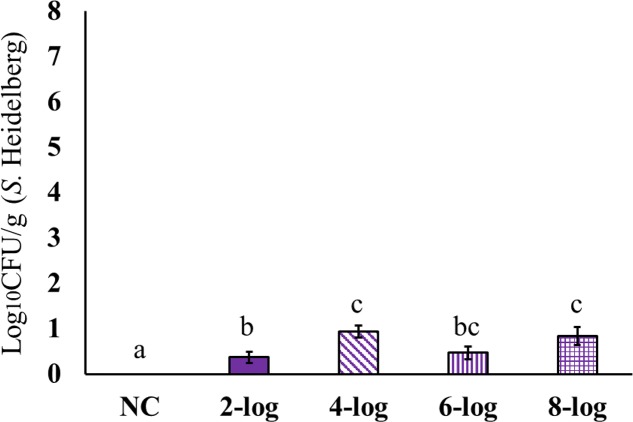
Effect of different inoculum levels on MDR *S.* Heidelberg deposition in the thigh (*Semimembranosus* muscle) of poults (Means ± SE; NC, 2-log, 4-log, 6-log, and 8-log treatments had a total of 20, 20, 18, 19, and 20 thigh samples/group, respectively, for the final analysis. ^a,b,c^ Bars with different superscripts differ significantly from each other at *P* < 0.05). NC, Negative Control.

**FIGURE 5:**
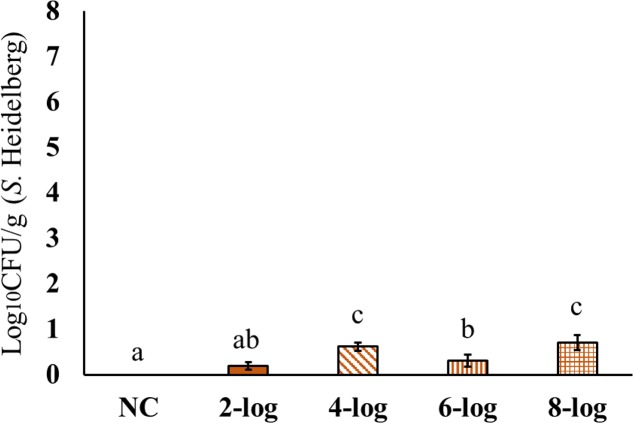
Effect of different inoculum levels on MDR *S.* Heidelberg deposition in the drumstick (*Peroneus longus* muscle) of poults (Means ± SE; NC, 2-log, 4-log, 6-log, and 8-log treatments had a total of 20, 20, 18, 19, and 20 drumstick samples/group, respectively, for the final analysis. ^a,b,c^ Bars with different superscripts differ significantly from each other at *P* < 0.05). NC, Negative Control.

**FIGURE 6:**
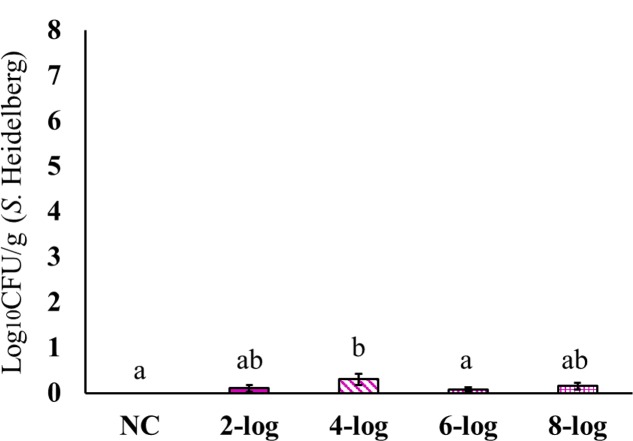
Effect of different inoculum levels on MDR *S.* Heidelberg deposition in the breast (*Pectoralis major* muscle) of poults (Means ± SE; NC, 2-log, 4-log, 6-log, and 8-log treatments had a total of 20, 20, 18, 19, and 20 breast samples/group, respectively, for the final analysis. ^a,b^ Bars with different superscripts differ significantly from each other at *P* < 0.05). NC, Negative Control.

Interestingly, MDR *S.* Heidelberg evidently reached muscle samples in poults (**Figures 4**–**6**). However, the deposition of the isolate was less in the muscles. The inoculum levels administered to the poults could not be related to the recovery of the pathogen from the muscle samples in both experiments. For different inoculum levels of *S.* Heidelberg, the pathogen recovery was 0.4–1.0 (**Figure 4**), 0.2–0.7- (**Figure 5**) and 0.1–0.3- (**Figure 6**) log_10_ CFU/g *S.* Heidelberg from thigh (49 positive samples /77 total), drumstick (41 positive samples /77 total), and breast muscles (18 positive samples /77 total), respectively.

This study revealed that even with a lower inoculum of 2-log *S.* Heidelberg, the colonization could reach ∼4 log_10_ CFU/g in the cecum of poults. This means that the ingestion of ∼100 *Salmonella* cells (2 log_10_ CFU/ml) could cause effective pathogen colonization in poults after 14 days of challenge. The results showed a significant dissemination of MDR *S*. Heidelberg to the internal organs, and resulted in muscle tissue deposition in poults. Both colonization and dissemination of *S.* Heidelberg in poults underscore the potential role of infected poults as sources of farm and product contamination. The situation warrants adoption of effective *Salmonella* intervention strategies starting at day 0. Appropriate hygienic measures have to be adopted to reduce the colonization between the flocks.

Previous studies have indicated *S.* Heidelberg as a colonizer in poultry similar to other predominant serovars such as *S.* Enteritidis. A survey carried out by Borsoi et al. (2011) found that *S.* Heidelberg colonized in the cecum of broiler chicks and appeared in the cecum after 6 h post-infection. After 12 h postinfection, *S.* Heidelberg counts were higher in the cecum compared to *S.* Enteritidis. To follow, after 72 h of infection, both the serovars showed similar colonization in the cecum. In the same study, *S.* Heidelberg showed similar invasion potential to the liver as that of *S.* Enteritidis. Similarly, Menconi et al. (2011) reported high colonization of *S.* Heidelberg in poults where 5 log_10_ CFU *S.* Heidelberg challenge resulted in ∼7.0 log_10_ CFU/g colonization in the cecum of poults. However, the same inoculum level resulted only in ∼3.0 log_10_ CFU/g colonization in the cecum of broiler chicks, indicating comparative propensity of the serovar for colonization in poults.

The high colonization potential and survival and multiplication of MDR *S.* Heidelberg in poultry could be attributed to its virulence mechanisms. *S.* Heidelberg possesses different transmissible plasmids that contain genes encoding antimicrobial resistance, virulence, and a VirB4/D4 type-IV secretion system. The plasmids having VirB4/D4 type-IV secretion system is unique to *S.* Heidelberg that promotes the invasion and prolonged survival in the intestinal epithelial cells and macrophages. In addition, the presence of VirB4/D4 type-IV secretion system increases the virulence of *S.* Heidelberg and enables the pathogen to down-regulate host immune system (Gokulan et al., 2013). The plasmids that encode resistance genes such as *bla*TEM-1, *aac (3)-IIa*, *aadA1*, *ant(3*_*)-Ia*, and *tetA* are poultry related plasmids which also accounts for increased virulence and survival of *S.* Heidelberg in poultry (Folster et al., 2012).

**FIGURE 7:**
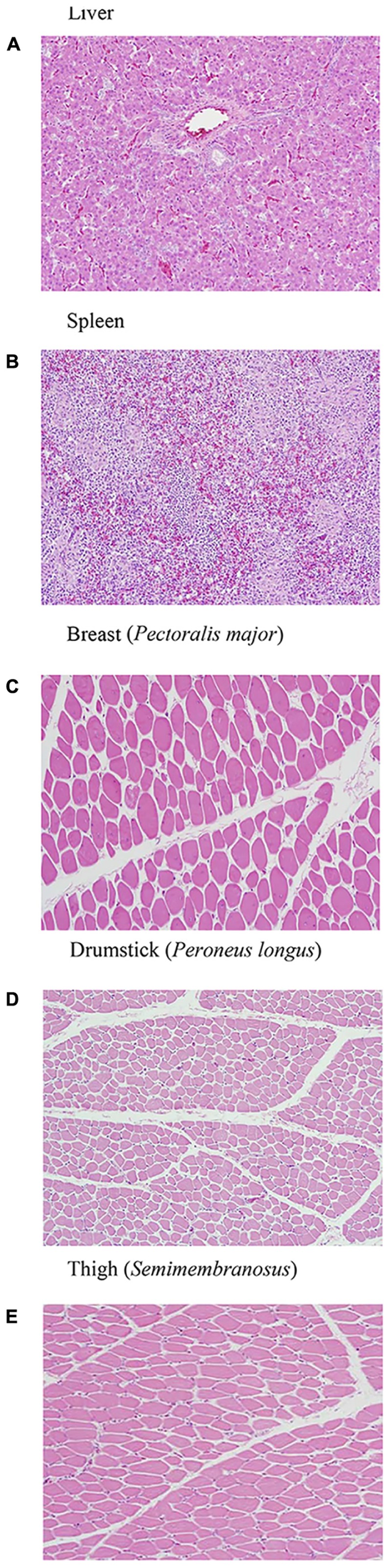
**(A–E)** H&E staining of liver, spleen, and muscle samples after 14 days of MDR *S.* Heidelberg challenge in poults did not reveal any pathological changes signifying an infection [only the representative samples from the highest inoculum (8 log_10_ CFU) are included].

**FIGURE 8:**
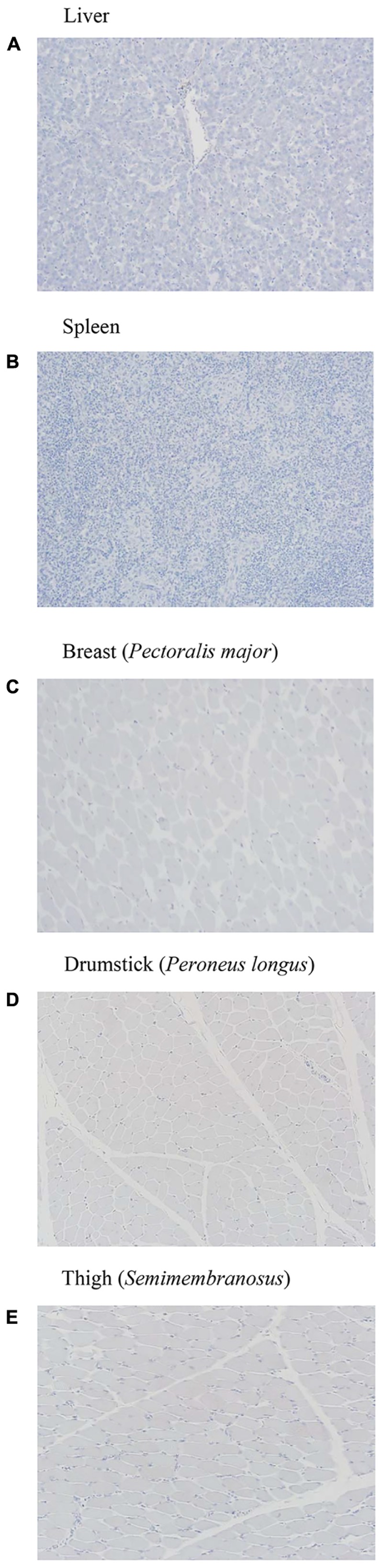
**(A–E)** Immunohistochemistry of liver, spleen, and muscle samples after 14 days of MDR *S.* Heidelberg challenge in poults did not reveal any pathological changes signifying an infection [only the representative samples from the highest inoculum (8 log_10_ CFU) are included].

In this study, the H&E and immunohistochemistry staining did not show pathological changes despite dissemination to liver, spleen or muscles (**Figures 7A–E**, **8A–E**). This could be because of the lower number of samples used for the analyses.

### Adult Turkey Hen Study

The recovery of *S.* Heidelberg from the cecum, internal organs (liver and spleen) and muscles (thigh, breast, and drumstick) of adult turkey hens 16 days after inoculation was determined. *S.* Heidelberg could be recovered from all *Salmonella* challenge groups in the cecum by surface plating method. In addition, recovery of MDR *S.* Heidelberg was assessed by enriching the samples for presence or absence the pathogen. The pathogen recovery rates from the cecum of adult turkey hens were 0, 50, 37.5, 50, and 62.5% respectively, for 0-, 2-, 4-, 6-, and 8-log inoculum levels (**Table 1**; *P* < 0.05 for linear dose-response). There was no recovery of *S.* Heidelberg from the liver samples from any of the groups (**Table 1**). Low recovery of MDR *S.* Heidelberg could be obtained from the spleen samples of adult turkey hens. No muscle deposition of MDR *S.* Heidelberg was noticed except in one drumstick sample in the 2-log inoculum group by the enrichment method. However, similar to the turkey poult study, the histology results did not reveal the potential presence of the pathogen (pictures not included) even though it was recovered from spleen, and muscle tissues by enrichment method at the higher inoculum level. In the present study, adult turkey hens seemed to be less susceptible to MDR *S.* Heidelberg infection at lower doses, as determined by surface plating. However, it should be noted that the birds were challenged at week 12. By this time, the turkeys would have developed a strong immune system and the protective natural microbiota (Smith and Tucker, 1980; Corrier et al., 1991).

**Table 1:** Effect of various inoculum levels of MDR *S.* Heidelberg on cecal colonization, dissemination to internal organs and deposition in skeletal muscles of adult turkey hens 16 days post inoculation (4 birds/group/study; 2 total studies).

Description	Experimental groups				
	0 CFU	10^∧^2 CFU	10^∧^4 CFU	10^∧^6 CFU	10^∧^8 CFU
Cecum^∗^	0/8 (0%)	4/8 (50%)	3/8 (37.5%)	4/8 (50%)	6/8 (62.5%)
Liver	0/8 (0%)	0/8 (0%)	0/8 (0%)	0/8 (0%)	0/8 (0%)
Spleen	0/8 (0%)	0/8 (0%)	0/8 (0%)	1/8 (12.5%)	2/8 (25%)
*Semimembranosus*	0/8 (0%)	0/8 (0%)	0/8 (0%)	0/8 (0%)	0/8 (0%)
*Peroneus longus*	0/8 (0%)	1/8 (12.5%)	0/8 (0%)	0/8 (0%)	0/8 (0%)
*Pectoralis major*	0/8 (0%)	0/8 (0%)	0/8 (0%)	0/8 (0%)	0/8 (0%)

*^∗^P-value for linear dose response < 0.05.*

Enrichment of the fecal samples from the isolation rooms was conducted daily for 16 days following inoculation of the adult turkey hens (**Table 2**). The fecal shedding of MDR *S.* Heidelberg was consistent in the challenge groups from days 1 to 11 post-inoculation, and all samples had MDR *S.* Heidelberg, indicating the constant shedding of the pathogen at all inoculum levels until day 11. This situation continued with the higher inoculum groups (10^∧^6, 10^∧^8) where almost all samples continued to be positive until the end of the study. The results indicated that when the level of inoculum is high, the possibility of shedding of the pathogen through the feces is high. However, for the lower inoculum levels also the excretion of the pathogen through the feces was detected throughout the study, although with differences noticed in the number of positive samples.

**Table 2:** Excretion level of MDR *S.* Heidelberg after challenging the adult turkey hens with different inoculum levels (by fecal enrichment; 2 samples/day/isolation room/study; total 2 studies).

	Experimental groups				
Days	0 CFU	10^∧^2 CFU	10^∧^4 CFU	10^∧^6 CFU	10^∧^8 CFU
1–11	0/4 (0%)	4/4 (100%)	4/4 (100%)	4/4 (100%)	4/4 (100%)
12	0/4 (0%)	2/4 (50%)	2/4 (50%)	4/4 (100%)	4/4 (100%)
13	0/4 (0%)	4/4 (100%)	0/4 (0%)	4/4 (100%)	4/4 (100%)
14	0/4 (0%)	4/4 (100%)	4/4 (100%)	4/4 (100%)	4/4 (100%)
15	0/4 (0%)	3/4 (75%)	2/4 (50%)	4/4 (100%)	3/4 (75%)
16^∗^	0/4 (0%)	1/4 (25%)	2/4 (50%)	4/4 (100%)	4/4 (100%)

*^∗^P-value for linear dose response < 0.05.*

## Conclusion

We demonstrated that MDR *S*. Heidelberg from the 2011 ground turkey outbreak was highly effective in colonizing poults, resulting in the dissemination of pathogen to liver and spleen, and showed potential for deposition in skeletal muscles of poults. The recovery rate of *S.* Heidelberg was highest in cecum followed by spleen, liver, thigh, drumstick, and breast. This is an important finding since most studies have not focused on the potential deposition of this pathogen in skeletal muscle tissues. The adult turkey hens, although positive for the pathogen in the cecum with all inoculum levels, responded well against the dissemination of *S*. Heidelberg in the liver and spleen, and potential deposition in the muscles. The results indicate that MDR *S*. Heidelberg has the capability for becoming a threat to the microbiological safety of turkeys and turkey products, warranting the producers to invest in targeted intervention methods to control it at the farm level. However, longer-duration challenge studies are warranted to determine if the pathogen inoculated at an earlier stage could potentially result in persisting colonization in the cecum, dissemination to the liver and spleen, and deposition in the muscles, posing a significant food safety threat to the industry.

## Author Contributions

DN, JV, and AK performed the turkey studies. RP read the histopathology and immunohistochemistry slides. DN and AK wrote the manuscript. JV, SN, and RP reviewed and corrected the manuscript before submission. SN helped AK to procure adult turkey hens from the commercial grower as a donation. AK conceived the idea, designed the study, and conducted the statistical analysis of the data.

## Conflict of Interest Statement

The authors declare that the research was conducted in the absence of any commercial or financial relationships that could be construed as a potential conflict of interest.
